# Multimode HMSIW-Based Bandpass Filter with Improved Selectivity for Fifth-Generation (5G) RF Front-Ends †

**DOI:** 10.3390/s20247320

**Published:** 2020-12-19

**Authors:** Amjad Iqbal, Jun Jiat Tiang, Sew Kin Wong, Mohammad Alibakhshikenari, Francisco Falcone, Ernesto Limiti

**Affiliations:** 1 Centre For Wireless Technology, Faculty of Engineering, Multimedia University, Cyberjaya 63100, Malaysia; amjad730@gmail.com (A.I.); jjtiang@mmu.edu.my (J.J.T.); skwong@mmu.edu.my (S.K.W.); 2 Electronic Engineering Department, University of Rome “Tor Vergata”, Via del Politecnico 1, 00133 Rome, Italy; limiti@ing.uniroma2.it; 3 Electrical Engineering Department, Public University of Navara, 31006 Pamplona, Spain; francisco.falcone@unavarra.es; 4 Institute of Smart Cities, Public University of Navarre, 31006 Pamplona, Spain

**Keywords:** substrate integrated waveguide (SIW), transmission zeros (TZs), metallic via, coupling topology

## Abstract

This article presents the detailed theoretical, simulation, and experimental analysis of a half-mode substrate integrated waveguide (HMSIW)-based multimode wideband filter. A third-order, semicircular HMSIW filter is developed in this paper. A semicircular HMSIW cavity resonator is adopted to achieve wide band characteristics. A U-shaped slot (acts as a λ/4 stub) in the center of a semicircular HMSIW cavity resonator and L-shaped open-circuited stubs are used to improve the out-of-band response by generating multiple transmission zeros (TZs) in the stop-band region of the filter. The TZs on either side of the passband can be controlled by adjusting dimensions of a U-shaped slot and L-shaped open-circuited stubs. The proposed filter covers a wide fractional bandwidth, has a lower insertion loss value, and has multiple TZs (which improves the selectivity). The simulated response of filter agrees well with the measured data. The proposed HMSIW bandpass filter can be integrated with any planar wideband communication system circuit, thanks to its planar structure.

## 1. Introduction

A wideband filter with high selectivity, low insertion loss, light weight, high quality factor, and high power handling capability is the key component for future wideband communication systems [1,2]. The substrate integrated waveguide (SIW) possesses most of the above requirements [3,4]. The operational principle of SIW is identical to a bulky waveguide, but SIW is a planar circuit, unlike a conventional waveguide. A planar structure of SIW provides an extra advantage when it is integrated with other planar circuits and system-in-package applications. Much more consideration is given to SIW technology for developing RF front-end components because of its several advantages over other technologies. A multimode filter is a type of filter that has more than one excited mode at once. So far, several multimode filters have been designed using planar SIW technology [5,6], multilayer resonators [7,8], and microstrip technology [9].

The wideband response is achieved by coupling together different resonators of different resonant frequencies. Several papers demonstrate the wideband performance by coupling nearby modes. In [10], a wideband filter using SIW technology is designed by coupling the modes securely with the help of U-shaped slots. The filter in this design has only 42% of the fractional bandwidth and is larger in size, using as many as five modes. In [11], a multi-mode wideband filter is designed using three modes of triangular SIW cavity. The wideband response is achieved by controlling the first resonant mode by means of an extra via hole of radius 0.5 mm in the corner of a triangular resonator. In [12], an SIW filter is designed for millimeter waves, where the length and width of the slots control the electric coupling and bandwidth of the filter. In [13], a wideband filter is designed using three modes of the circular SIW cavity. The coupling between the modes is controlled by putting an extra via hole in the center of the resonator. Degenerated modes in the bandpass filter are used in [14] to achieve wideband. Bandpass filters, having this topology, achieve relatively small sizes with low fractional bandwidth. In [15], a UWB bandpass filter, based on a square-shaped defected ground plane and microstrip resonators, is designed. It covers the whole UWB band (3.1–10.6 GHz). The operating bandwidth of the filter is enhanced by adding two short-circuited stubs. Then, an open-circuited stub is introduced to generate a notch (stopband) at 5.8 GHz. In [16], a UWB bandpass filter with a notch band is reported. The reported filter has three layers: the lower and upper layers have T-shaped microstrip resonators, and the middle layer has three circular slots for coupling the lower and upper layers. Multiple modes of the microstrip resonators are excited to obtain a large bandwidth. Then, the microstrip resonators and ground plane are modified to get a notch band at 5.8 GHz. In [17], a wideband filter is reported. The wideband response is achieved by tight coupling between the resonators. In addition, the source/load coupling with the resonators is also strong, which leads to a wideband bandpass filter. In [18], different filters for dual- and triple-band 5G applications are designed. The reported filters use open-loop SIRs for dual-band operation and uniform folded resonators for triple-band applications. The reported filters have wideband characteristics in each passband with good out-of-band rejections. The authors of [19] reported a wideband bandpass filter. The reported filter is designed by using square ring resonators, which are loaded by stubs. In [20], wideband second- and third-order bandpass filters are designed using open- and short-circuited stubs. The reported filter has a wideband (51.9% in the lower-frequency passband and 23.3% in the high-frequency passband) and low insertion loss of 0.3 dB.

In the present paper, a circular half-mode SIW (HMSIW) resonator is used to design a wideband filter. The wideband response is achieved by coupling the first three modes. In addition, the selectivity is enhanced by generating the source-load coupling topology using a U-shaped slot and L-shaped open-circuited stub resonators.

## 2. Full-Mode SIW and Half-Mode SIW Cavity Resonator Analysis

**Figure 1:**
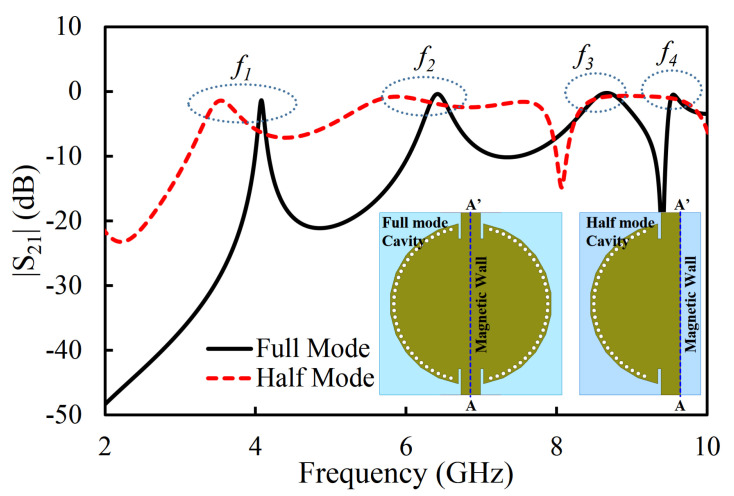
Transmission coefficient (S_21_) (magnitude) of the filter in the full-mode and half-mode substrate integrated waveguide (SIW) cavity resonator.

**Figure 2:**
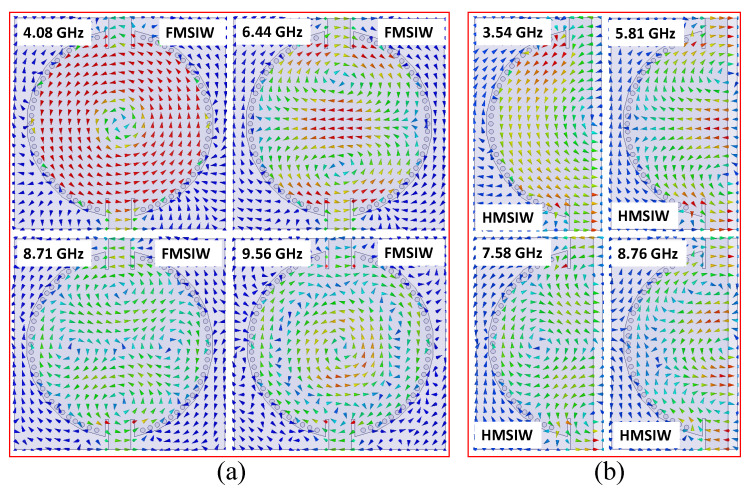
*H*-fields plot of the first four modes in the (**a**) full-mode circular SIW resonator, (**b**) half-mode circular SIW resonator.

A circular SIW cavity resonator with a diameter of 40 mm is suggested, as depicted in Figure 1. The initial dimensions of the circular SIW cavity are obtained with the help of Equation (1) [21] and then optimized using a full-wave electromagnetic (EM) simulator (high-frequency structure simulator; HFSS). The circular cavity is developed by placing metallic vias on the edges. The radius (*r*) and isolation among the plated holes (*d*) are selected according to the guidelines (2*r*/*d* ≥ 0.5, and 2*r*/λ ≤ 0.1) suggested in [22], so that minimal power leaks from the cavity. RT/duroid 5880 ($\epsilon_{r}$ = 2.2) is used as a substrate. The width of the transmission line ($W_{tl}$) is calculated using standard transmission line equation for 50 Ω impedance [21]. The transmission coefficient (|S_21_|) of the full-mode SIW (FMSIW) is plotted in Figure 1. The first four resonant modes (TM_11_, TM_21_, TM_22_, and TM_33_) are labeled $f_{1}$ to $f_{4}$, with $f_{1}$ as the lowest-frequency mode followed by the higher-frequency modes as far as $f_{4}$. The resonant frequencies of the first four resonant modes of the circular SIW cavity resonator are located at 4.08 GHz (TM_11_ mode), 6.44 GHz (TM_21_ mode), 8.71 GHz (TM_22_ mode), and 9.56 GHz (TM_33_ mode). The FMSIW cavity resonator is converted to a HMSIW cavity resonator by cutting along A and A′ (quasi-magnetic wall), as shown in Figure 1. The A-A′ line on the open-ended side of the cavity is a quasi perfect magnetic boundary. The |S_21_| of the HMSIW cavity resonator is also illustrated in Figure 1. The first four resonant modes of the HMSIW cavity resonators lie at 3.54 GHz (TM_11_ mode), 5.81 GHz (TM_21_ mode), 7.58 GHz (TM_22_ mode), and 8.76 GHz (TM_33_ mode). A shift towards the lower-frequency side is observed in the resonant modes of the HMSIW cavity resonator as compared to the FMSIW cavity resonator. The lower-frequency shift in the HMSIW cavity resonator is due to the fringing effects caused by the open-ended magnetic wall. The *H*-field plots of the first four modes in the FMSIW and HMSIW cavity resonators are shown in Figure 2a,b, respectively.

(1) $$f_{r}=\frac{k_{mnp}c}{2\pi r\sqrt{\epsilon_{reff}}}$$

where *c* is the speed of light in the free space, and $\epsilon_{reff}$ is the effective permittivity of the substrate. The radius of the cavity resonator is denoted by *r*, the resonant frequency is denoted by $f_{r}$, and the value of $k_{mnp}$ can be computed by substituting Bessel’s coefficients.

## 3. HMSIW Wideband Bandpass Filter Design

**Figure 3:**
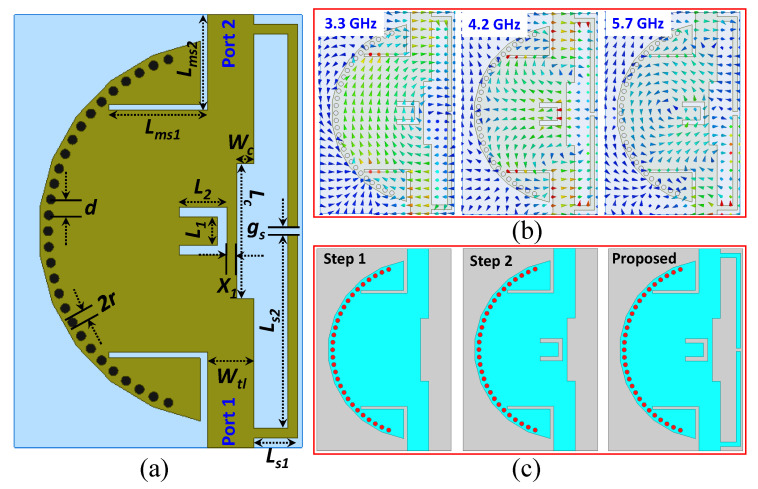
(**a**) Geometry of the proposed wideband filter (*d* = 1.65, 2r = 1, $W_{tl}$ = 5, $L_{s1}$ = 4.5, $L_{s2}$ = 21.1, $g_{s}$ = 0.8, $L_{c}$ = 14, $W_{c}$ = 2, $L_{1}$ = 2, $L_{2}$ = 6, $L_{ms1}$ = 10.3, $L_{ms2}$ = 9.3, $X_{1}$ = 2 (Unit = mm)); (**b**) *H*-fields plot at 3.3, 4.2, and 5.7 GHz; (**c**) design evolution steps [1].

Figure 3a shows the design of the proposed HMSIW cavity resonator-based multimode wideband filter. The optimized dimensions can be found in the caption of Figure 3a. A rectangular inductive matching slot is designed with inset feeding to further improve the impedance matching of the filter. Therefore, the proposed filter has three excited resonant modes: TM_11_ at 3.3 GHz, TM_21_ at 4.2 GHz, and TM_22_ at 5.7 GHz, as shown in Figure 3b. The design procedure of the proposed filter, which consists of three iterations, is illustrated in Figure 3c. Initially, a semi-circular HMSIW cavity resonator is designed. Then, the filter is modified by loading a U-shaped slot; as a result, the TZ on the high-frequency side of the passband is generated, and selectivity is enhanced. Finally, in addition, two L-shaped stubs are added to further improve the selectivity. As a result, two TZs are generated on the lower-frequency side of the passband. Simulation of the proposed filter is carried out using full-wave EM simulator HFSS (solution type = driven model, frequency range = 1–12 GHz, step size = 1 MHz, type of sweep = discrete, maximum number of passes = 16, maximum delta energy = 0.05, type of ports = wave-ports, and boundary condition = radiating only in all direction). The proposed structure is designed, and wave-ports, having 50 Ω impedance, at both ends of the transmission line are assigned. A detailed design and analysis of each iteration is discussed in the following sections.

### 3.1. Step 1: Wideband Filter Design with No Transmission Zero (TZ)

**Figure 4:**
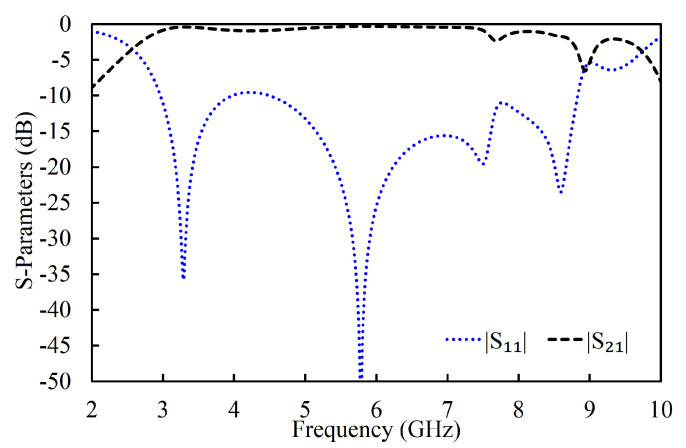
Simulated S-parameters (magnitude) of the filter in the first iteration [1].

The maximum fields of the first four modes lie near to the arbitrary magnetic wall of the HMSIW cavity resonator (see Figure 2b). Therefore, a rectangular slot at the maximum fields side of the HMSIW cavity resonator was introduced to adjust the positions of the resonant modes. The reflection coefficient (|S_11_|) and transmission coefficient (|S_21_|) of the filter in the first iteration are shown in Figure 4. It can be observed that the passband is generated by the four coupled resonant modes.

**Figure 5:**
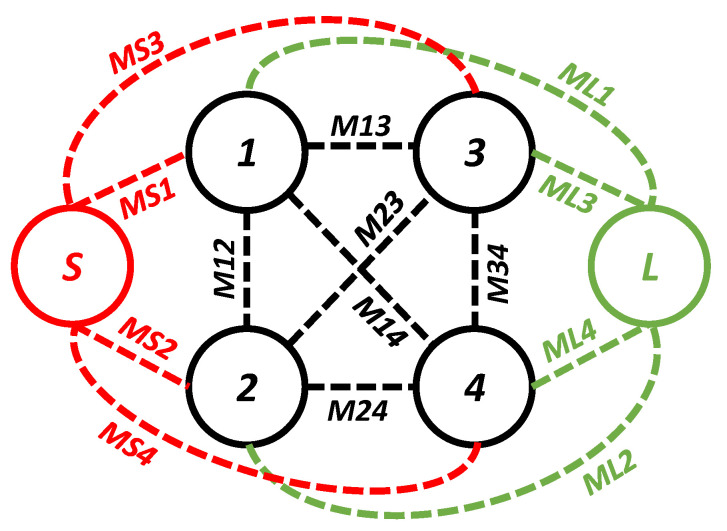
Coupling topology of the filter in the first iteration.

**Figure 6:**
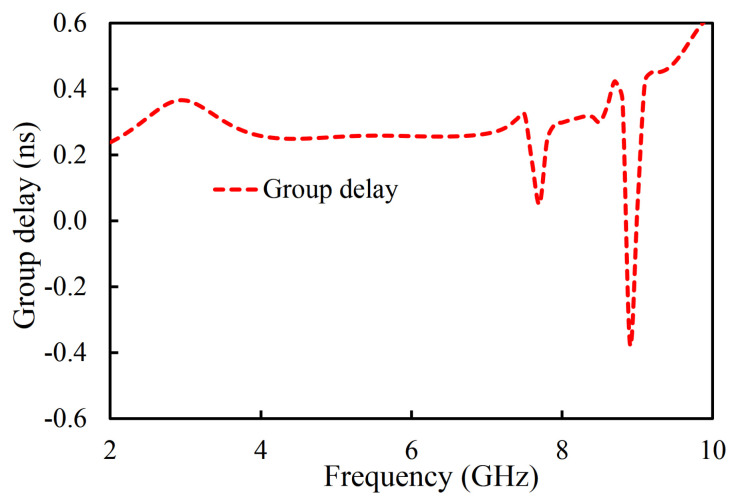
Simulated group delay of the filter in the first iteration and coupling topology.

During this iteration, the first four resonant modes created a passband. The coupling topology of the filter in the first iteration is shown in Figure 5. The coupling between the source and the first mode is *MS1*; between the source and the second mode it is *MS2*; between the source and the third resonator it is *MS3*; and between the source and the fourth mode it is *MS4*. From the load to the first mode it is *ML1*; from the load to the second mode it is *ML2*; from the load to the third resonator it is *ML3*; and from the load to the fourth mode it is *ML4*. From the first and second mode it is *M12*; from the first and third mode it is *M13*; from the second and third mode it is *M23*; from the second and fourth mode it is *M24*; and from the third and fourth mode it is *M34*. The passband filter, designed in the first iteration, has four poles located at 3.29, 5.77, 7.5, and 8.6 GHz. An unwanted dip in the |S_21_| graph of the filter at the passband can be seen at 7.7 GHz. In addition, a 3 dB fractional bandwidth of 117% at the center frequency of 6.12 GHz is noted. Moreover, the designed filter (first iteration) has a large bandwidth but is not suitable for applications where high selectivity is required. The selectivity of the filter can be enhanced by introducing the source-load coupling topology. The simulated group delay varies from 0.04 ns to 0.58 ns in the passband (2.51–9.73 GHz), as shown in Figure 6.

**Figure 7:**
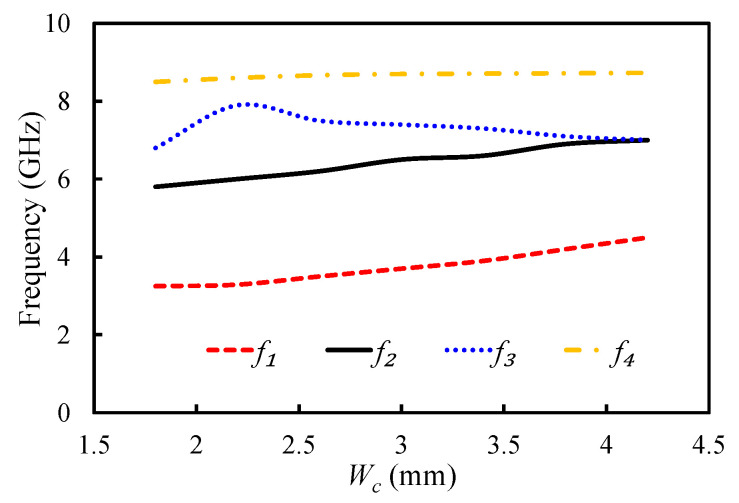
Mode chart for the first four resonant modes against the varying $W_{c}$.

**Figure 8:**
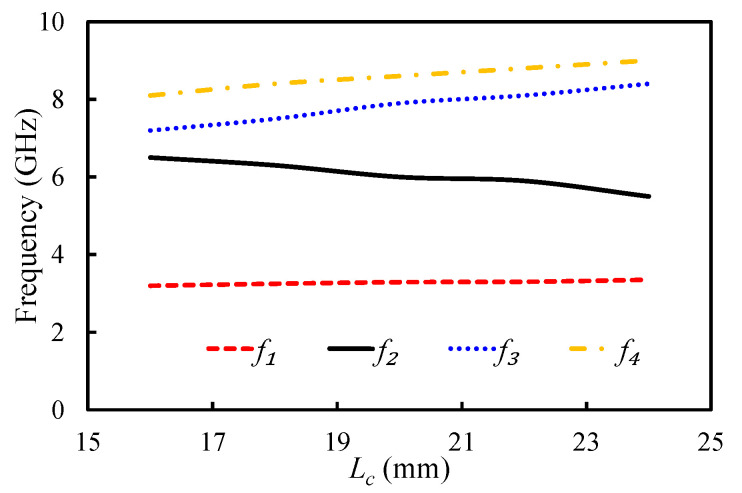
Mode chart for the first four resonant modes against the varying $L_{c}$.

The positions of the resonant frequencies of the first four modes of the HMSIW filter were analyzed by changing $W_{c}$ and $L_{c}$ parameters of the rectangular slot. The impact of the changing $W_{c}$ and $L_{c}$ on the position of the resonant modes is illustrated in Figure 7 and Figure 8. $f_{1}$, $f_{2}$, $f_{3}$, and $f_{4}$ show the resonant frequencies of the first mode (TM_11_ mode), second mode (TM_21_ mode), third mode (TM_22_ mode), and fourth resonant mode (TM_33_ mode), respectively. By changing the $W_{c}$ value from 1.8 to 4.2 mm, the first resonant mode shifted from 3.25 to 4.5 GHz; the second resonant mode shifted from 5.8 to 7 GHz; and that of the fourth resonant mode shifted from 8.5 to 8.73 GHz. The third resonant mode had an irregular relationship with the $W_{c}$, as illustrated in Figure 7. The dependency of the first four resonant modes on the slot length ($L_{c}$) is presented in Figure 8. The positions of $f_{1}$, $f_{3}$, and $f_{4}$ were directly related to the $L_{c}$, and that of $f_{2}$ was inversely related to the $L_{c}$, as illustrated in Figure 8. The resonant frequencies of the first four modes were located at 3.2, 6.5, 7.2, and 8.1 GHz, when $L_{c}$ was 16 mm. At $L_{c}$ = 24 mm, the resonant frequencies of the first four modes shifted to 3.5, 5.5, 7.2, and 9 GHz. It can be observed that these parameters played a key role in positioning the first four resonant modes. All four resonant modes can be coupled together, by properly adjusting these parameters, to get a wide passband.

### 3.2. Step 2: Wideband Filter Design with One TZ

As can be seen from Figure 4, the filter had poor selectivity in the first iteration. In order to enhance the selectivity of the filter, a U-shaped slot was etched on the upper metal layer of the HMSIW cavity resonator. The structure of the filter in the second iteration is displayed in Figure 3c. The dimensions of the U-shaped slot ($L_{1}$ and $L_{2}$) are derived using Equation (2). The slot in the resonator plays the role of a λ/4 stub, which generates transmission zero on the higher-frequency side of the passband. Therefore, the selectivity of the filter was enhanced on the higher-frequency side of the passband. As a result, a TZ on the higher-frequency side of the passband was generated.

(2) $$f_{z}=\frac{c}{2L\sqrt{\epsilon_{reff}}}$$

In the above equation, $f_{z}$ is the frequency of the TZ, *L* (*L* = $L_{1}$ + $L_{2}$) is the total length of the slot, *c* is the speed of light in a vacuum, and $\epsilon_{reff}$ is the effective dielectric constant of the substrate.

**Figure 9:**
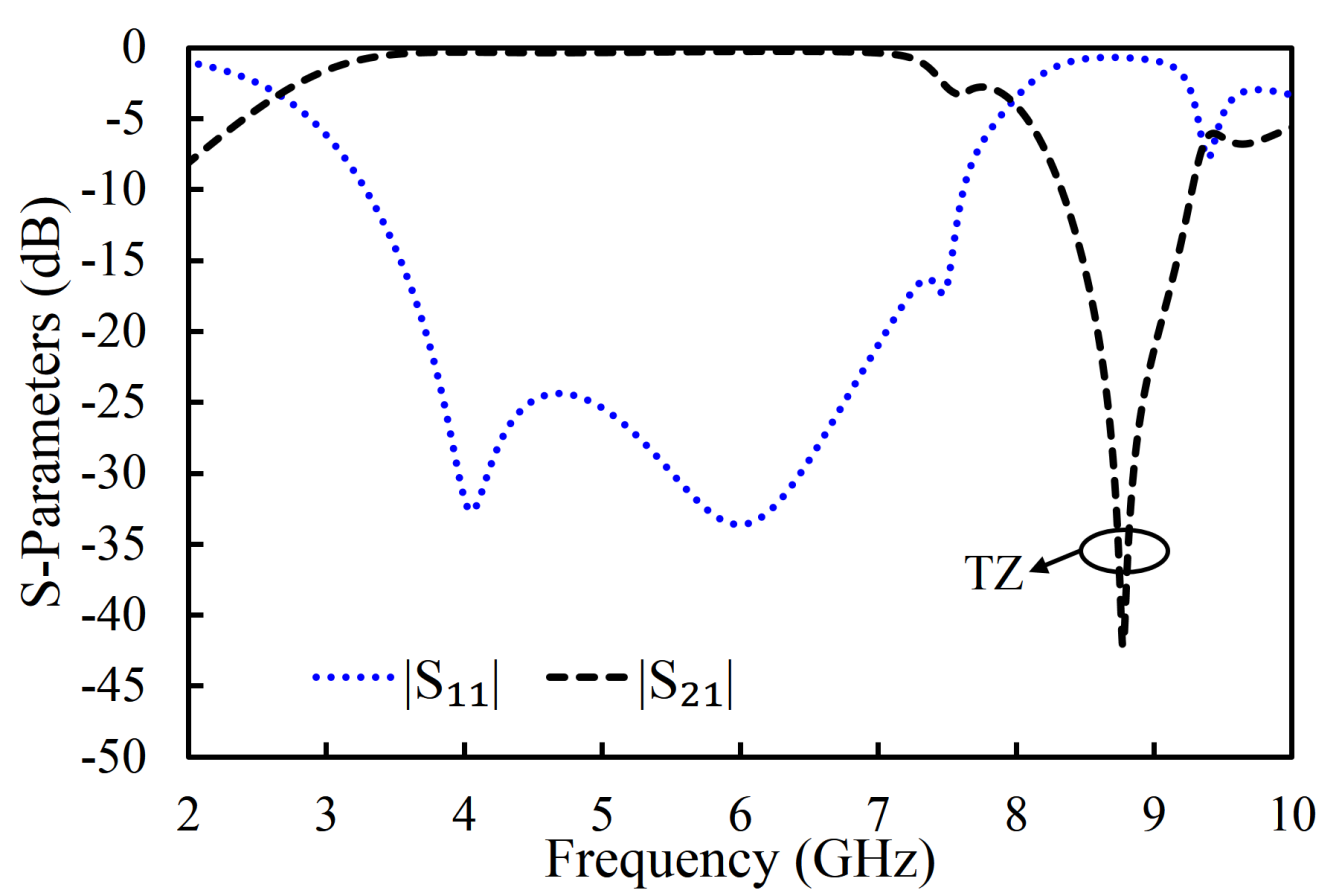
Simulated S-parameters (magnitude) of the filter in the second iteration [1].

**Figure 10:**
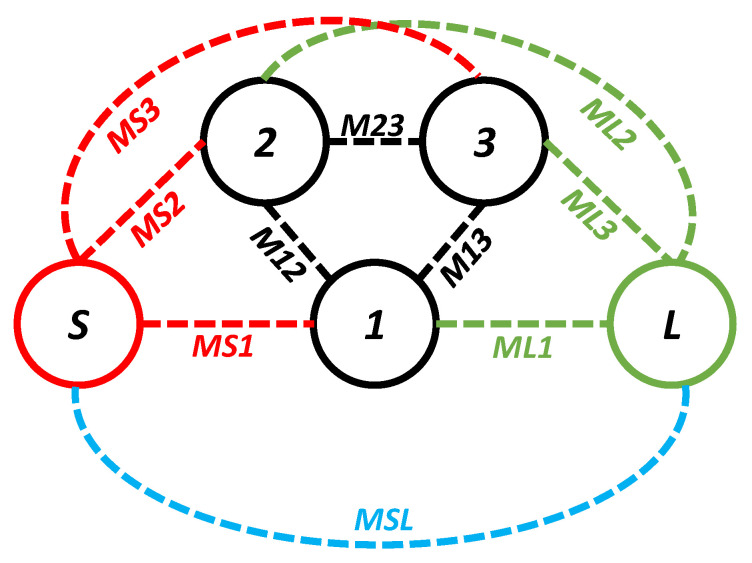
Coupling topology of the filter in the second iteration.

**Figure 11:**
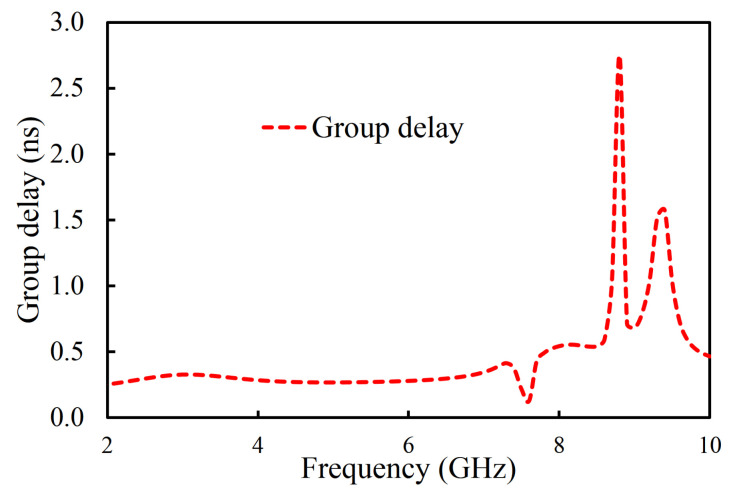
Simulated group delay of the filter in the second iteration.

The |S_11_| and |S_21_| of the filter in the second iteration are illustrated in Figure 9. Only the first three resonant modes reached the passband of the filter, as shown in Figure 9. The designed filter in the second iteration had its first pole at 4.08 GHz, the second pole at 6.06 GHz, and the third pole at 7.48 GHz. One TZ, generated due to the introduction of the U-shaped slot, was noted at 8.78 GHz. As a result, a 3 dB fractional bandwidth of 94% at the center frequency of 5.11 GHz was observed. The filter, designed in the second iteration, had a lower 3 dB bandwidth than the filter in the first iteration. However, the filter in the second iteration had improved selectivity at the higher-frequency side of the passband than the filter in the first iteration. The coupling topology of the filter is shown in Figure 10. The source-load coupling, which is responsible for the sharper selectivity on the higher frequency side of the passband, is obvious in the coupling topology. The simulated group delay varied from 0.25 ns to 0.41 ns in the passband (2.71–7.52 GHz), as illustrated in Figure 11.

**Figure 12:**
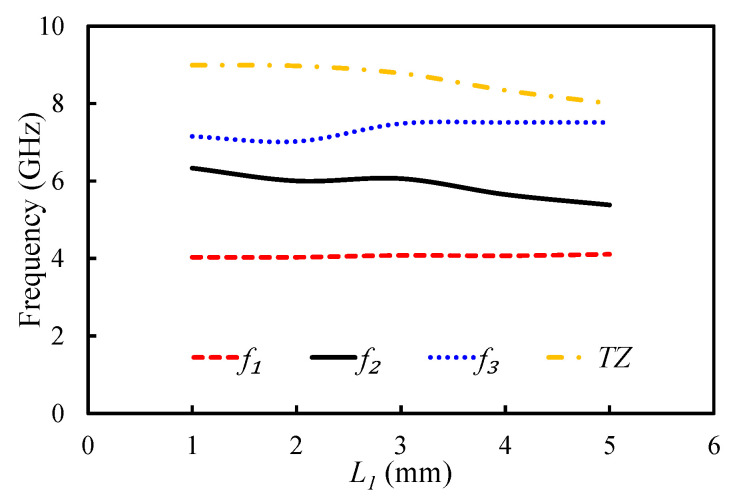
Mode chart for the first three resonant modes and a transmission zero (TZ) against the varying $L_{1}$.

**Figure 13:**
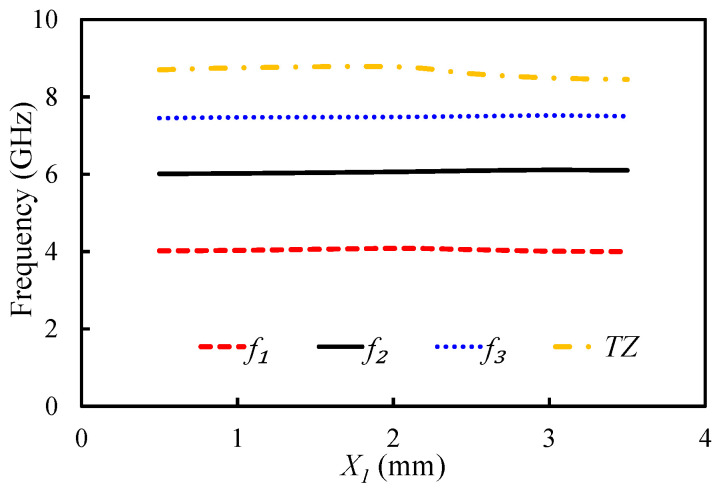
Mode chart for the first three resonant modes and a TZ against the varying $X_{1}$.

The impact of parameters $L_{1}$ and $X_{1}$ on the position of the first three resonant modes and TZ was analyzed as shown in Figure 12 and Figure 13. As Equation (2) clearly shows, the slot length is related to the position of TZ. The effective length of the slot has a inverse relationship with the TZ: the TZ shifts to the lower-frequency side with an increase in the dimensions of the U-shaped slot. In fact, the position of TZ can be controlled by adjusting the length of the U-shaped slot. Figure 12 shows the impact of $L_{1}$ on the first three resonant modes and the TZ. The TZ of the filter shifted from 8.99 to 8 GHz by changing the value of $L_{1}$ from 1 to 5 mm. The selection of a suitable length for $L_{1}$ in terms of selectivity and bandwidth is crucial. If we increase $L_{1}$ to a certain extent, then the TZ can appear before the third mode. As a result, the operating bandwidth will reduce. Moreover, no significant change on the position of the first resonant mode was observed with the $L_{1}$. However, the position of the second and third resonant modes changed with the $L_{1}$. Changing the value of $L_{1}$ from 1 to 5 mm made the first resonant mode shift from 4.03 to 4.1 GHz; the second mode shifted from 6.33 to 5.38 GHz; and the third mode shifted from 7.15 to 7.51 GHz. The position of the U-shaped slot had less impact on the resonant modes and TZ, as shown in Figure 13. When $X_{1}$ was changed from 0.5 to 3.5 mm, the TZ shifted from 8.7 GHz to 8.45 GHz.

### 3.3. Proposed Filter: Wideband Filter with Enhanced Selectivity

**Figure 14:**
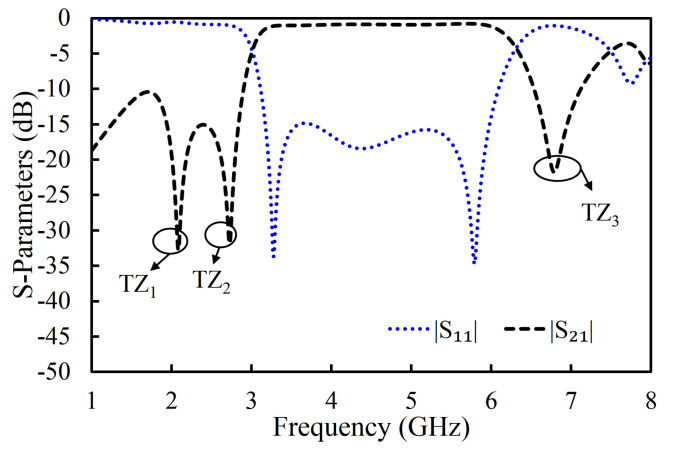
Simulated S-parameters (magnitude) of the filter in the proposed filter [1].

**Figure 15:**
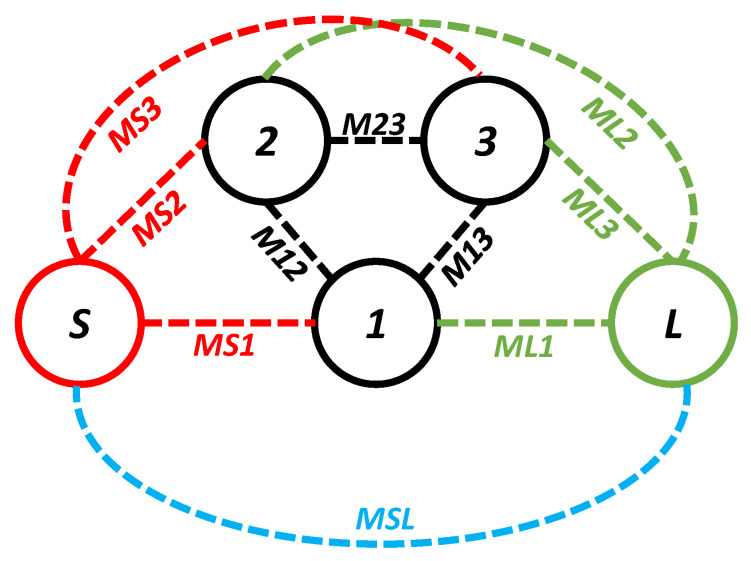
Coupling topology of the proposed filter.

**Figure 16:**
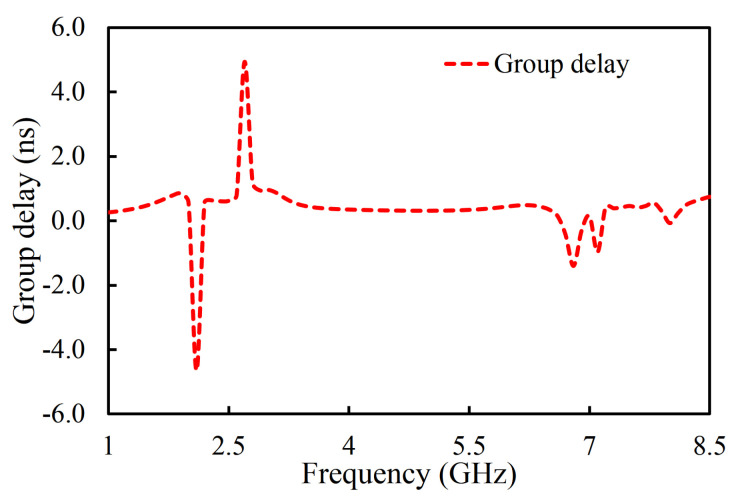
Simulated group delay of the filter in the proposed filter.

In the third iteration, two L-shaped open-circuit stub resonators were connected to both the source and the load in order to further enhance selectivity, as shown in Figure 3c. As a result, two TZs were generated on the lower-frequency side of the passband because of the additional source-load coupling caused by the open-circuited stubs. The |S_11_| and |S_21_| of the proposed filter are shown in Figure 14. The proposed filter has three TZs: two on the lower-frequency side of the passband and one on its higher-frequency side. The TZs are located at 2.09, 2.72, and 6.82 GHz with respective attenuation levels of −33, −32.4, and −22.1 dB. The proposed filter has three poles, which are located at 3.3, 4.29, and 5.75 GHz. Moreover, the filter designed in the third iteration has a 3 dB fractional bandwidth of 69.31% at the center frequency of 4.67 GHz. The coupling topology of the filter is displayed in Figure 15. The simulated group delay varied from 0.31 to 0.8 ns in the passband (3.05–6.29 GHz), as shown in Figure 16. It can be observed that the filter had a small group delay in the passband; however, the group delay increased where the TZs were located. The large group delay values (peaks) in the group delay plots indicate the presence of the TZs.

**Figure 17:**
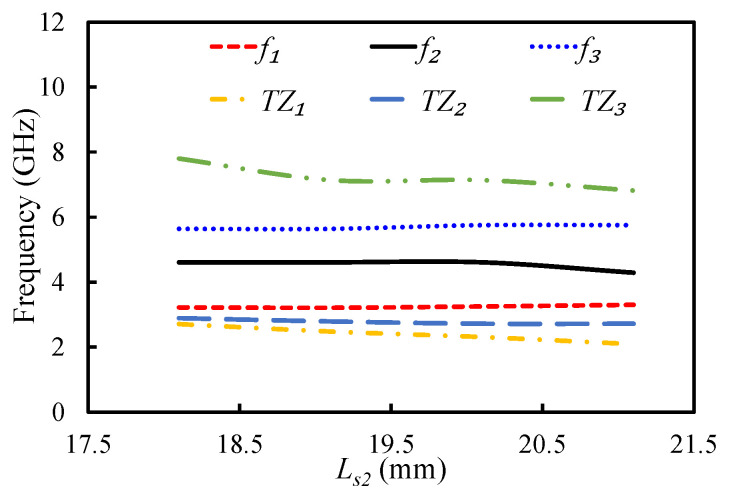
Mode chart for the first three resonant modes and transmission zeros against $L_{s2}$.

**Figure 18:**
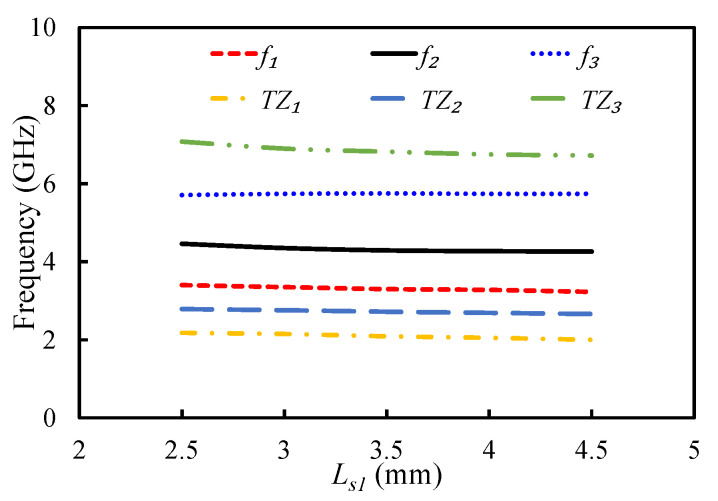
Mode chart for the first three resonant modes and transmission zeros against $L_{s1}$.

Figure 17 and Figure 18 display the impact of varying parameters $L_{s2}$ and $L_{s1}$ on the first three resonant modes and the TZs of the filter. By increasing the length of $L_{s2}$, the coupling between the open-circuited stubs increased; hence, the first two TZs moved further apart, as illustrated in Figure 17. The position of the third TZ shifted to the lower-frequency side when $L_{s2}$ was increased. The resonant frequency of the second mode shifted to the lower-frequency side by increasing the $L_{s2}$, while the first and third modes were resistant to any change in $L_{s2}$. The parameter $L_{s1}$ had a negligible effect on the first three resonant modes and the first two TZs, but it had an inverse relationship with the third TZ. Therefore, $L_{s2}$ and $L_{s1}$ can be adjusted to position the TZs of the filter.

## 4. Results and Discussion

**Figure 19:**
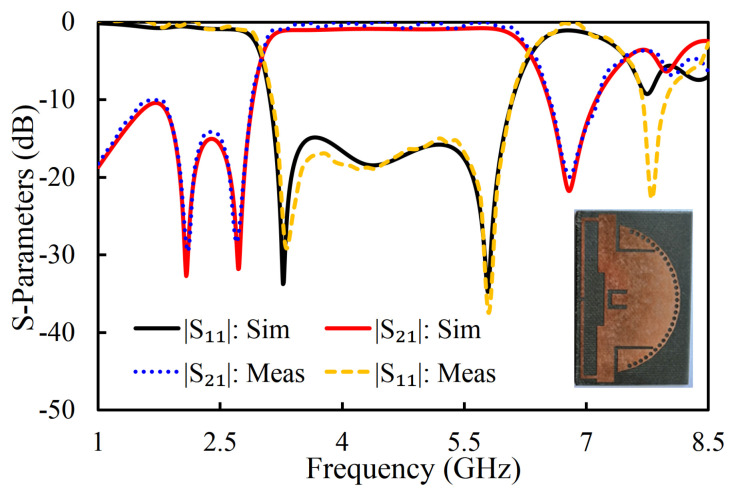
Simulated and measured S-parameters (magnitude) of the proposed filter.

**Figure 20:**
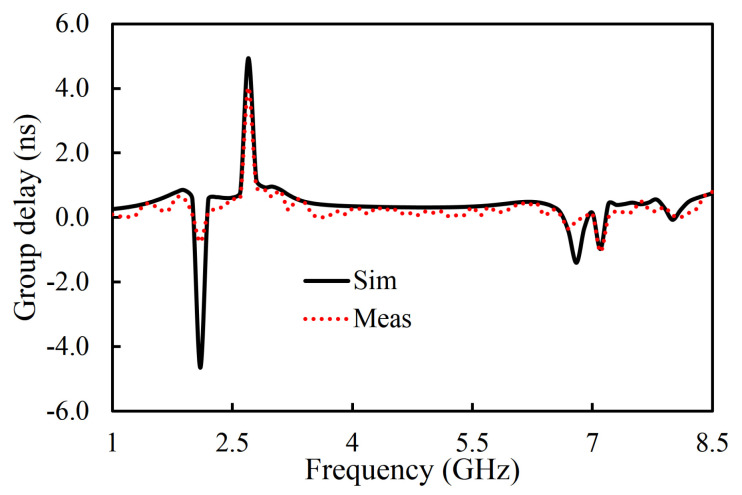
Simulated and measured group delay of the filter.

**Figure 21:**
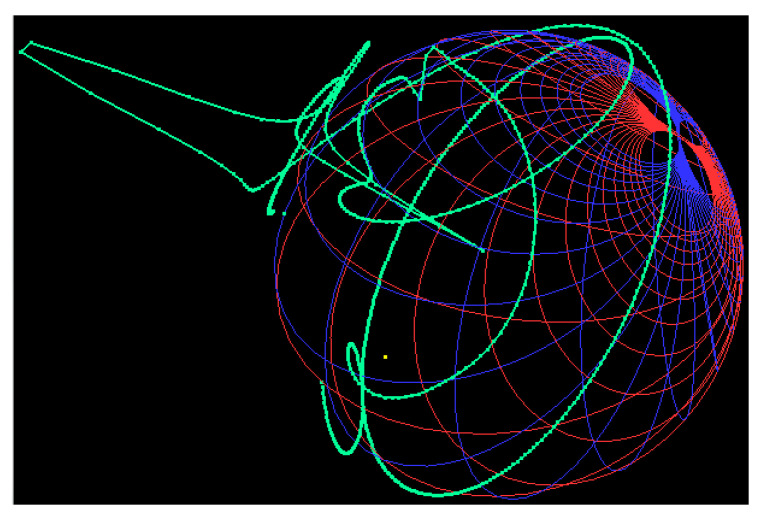
Group delay of the proposed filter over a 3D Smith chart.

**Figure 22:**
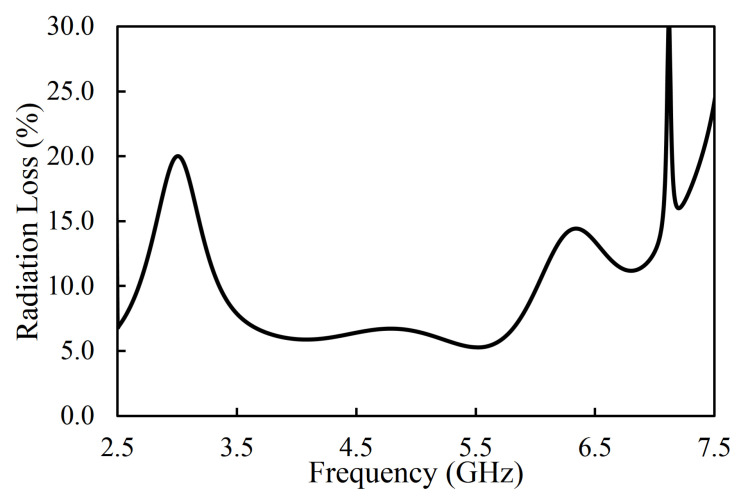
Simulated radiation loss of the filter.

The fabricated prototype of the proposed filter is shown in Figure 19. RT/duroid 5880 ($\epsilon_{r}$ = 2.2, loss tangent of 0.0009, and thickness of 1.575 mm) was used as a substrate. The performance of the fabricated filter was measured with the Network Analyzer. The S-parameter results of the fabricated filter were compared with the simulation ones, as shown in Figure 19. The measured center frequency was 4.66 GHz with a fractional bandwidth of 67.8%. It can be seen that the insertion loss was better than 1 dB in the whole passband. The TZs were located at 2.09, 2.72, and 6.82 GHz, as shown in Figure 19. The measured group delay of the filter varied from 0.09 ns to 0.81 ns, as shown in Figure 20. The group delay of the filter is plotted over a 3D Smith chart [23]. The above region from the surface is a positive peak of the group delay, and the interior region from the surface is a negative peak, as shown in Figure 21. The comparison between simulation and measurement results shows good agreement, with differences on the order of 1 dB, on average, in the case of S_11_ parameters. The observed deviations are given mainly by connector effects, specifically to losses as well as reactive loading inherent to the connector soldering process. Radiation loss may result from etching the U-shaped slot on the upper surface of the HMSIW resonator. Therefore, the forward radiation loss ($RL_{fl}$) was calculated using Equation (3) [6]. The radiation loss was <10% in the whole operating band, as illustrated in Figure 22.

(3) $$RL_{fl}=1-|S_{11}{|}^{2}-{\left|S_{21}\right|}^{2}$$

Table 1 compares the proposed filter with the state-of-the-art wideband filters. The proposed filter has a higher fractional bandwidth than any other filter. The insertion loss of the filter is better than the other filters, except [11]. However, [11] has a lower fractional bandwidth and fewer TZs.

Based on the above studies, the following guidelines are suggested.

1. Select the dimensions of the cavity, based on the design specifications of the filter, using the following equation:
  $$f_{r}=\frac{k_{mnp}c}{2\pi r\sqrt{\epsilon_{reff}}}$$
2. Select the radius of the vias and gap between them as suggested by [22]. The suggested guidelines are *d*/*a* ≥ 0.5 and *d*/$\lambda_{\circ }$ ≤ 0.1 (where $\lambda_{\circ }$ is the wavelength at the center frequency, *d* is the diameter of the vias, and *a* is the gap between the vias).
3. Symmetrically cut the circular SIW cavity resonator into two portions. Each part is the HMSIW cavity resonator.
4. Adjust the dimensions of the open-ended slot ($L_{c}$ and $W_{c}$) to position the resonant modes of the cavity.
5. Design a U-shaped slot in the center of the cavity resonator to generate a TZ on the higher-frequency side of the passband. The following equation can be used to estimate the dimensions of the slot:
  $$f_{z}=\frac{c}{2L\sqrt{\epsilon_{reff}}}$$
6. Adjust the dimensions of the slot ($L_{1}$, and $L_{2}$) and position of the slot ($X_{1}$) to control the location of the TZ. Moreover, these parameters are also vital for positioning the resonant modes.
7. To further improve the selectivity, add L-shaped open-circuited stubs to generate the TZs on the lower-frequency side of the passband.
8. Adjust the dimensions of the L-shaped open-circuited stubs ($L_{s1}$, and $L_{s2}$) to control the location of the resonant modes and TZs.
9. Optimize the overall filter parameters to get the desired results.

## 5. Conclusions

A multimode wideband HMSIW filter with a fractional bandwidth of 69.31% at a center frequency of 4.67 GHz, return loss better than 15 dB, and insertion loss of <0.9 dB was designed and experimentally validated in this paper. The proposed filter was fabricated on the RT/duroid 5880 ($\epsilon_{r}$ = 2.2, loss tangent of 0.0009, and thickness of 1.575 mm) substrate. The measured results show good agreement with the simulation in terms of S-parameters and group delay. The proposed HMSIW bandpass filter can be integrated with any planar wideband communication system circuit, thanks to its planar structure. It covers the majority of fifth-generation (5G) sub-6 GHz bands (USA: 3.3–3.8 GHz, 4–5 GHz; Japan: 3.6–5 GHz; and South Korea, Taiwan, China, Russia, India, Australia, and EMEA: 3.3–3.8 GHz). The proposed filter is promising choice for next-generation wireless technologies (5G) due to its low insertion loss, competitive in band return loss, wide bandwidth, and good selectivity.

## Figures and Tables

**Table 1:** Comparison with the published wideband filters.

Ref.	Size ($\lambda_{\circ }\times \lambda_{\circ }$)	FBW (%)	$f_{\circ }$ (GHz)	IL (dB)	TZs
**This Work**	**0.69 × 1.03**	**69.31**	**4.67**	**0.9**	**3**
[6]	0.22 × 1.2	29	3.45	1.5	0
[7]	Not Given	10.34	5.8	1.1	3
[9]	≈0.58 × 0.91	69.1	4.5	1.4	2
[10]	0.63 × 1.25	42	8.5	1.1	3
[11]	Not Given	38	5.2	0.74	2
[14]	1.48 × 0.39	27.2	1	1.62	4

$\lambda_{\circ }$ = free space wavelength at the center frequency ($f_{\circ }$), IL = insertion loss; FBW = fractional bandwidth
